# Quantification of Chemical Uptake into the Skin by Vibrational Spectroscopies and Stratum Corneum Sampling

**DOI:** 10.1021/acs.molpharmaceut.2c01109

**Published:** 2023-04-13

**Authors:** M. Alice Maciel Tabosa, Pauline Vitry, Panagiota Zarmpi, Annette L. Bunge, Natalie A. Belsey, Dimitrios Tsikritsis, Timothy J. Woodman, K.A. Jane White, M. Begoña Delgado-Charro, Richard H. Guy

**Affiliations:** † Department of Life Sciences, University of Bath, Claverton Down, Bath BA2 7AY, U.K.; ‡ Department of Chemical and Biological Engineering, Colorado School of Mines, Golden, Colorado 80401, United States; § Chemical and Biological Sciences Department, National Physical Laboratory, Teddington TW11 0LW, U.K.; ∥ Department of Chemical and Process Engineering, University of Surrey, Guildford GU2 7XH, U.K.; ⊥ Department of Mathematical Sciences, University of Bath, Claverton Down, Bath BA2 7AY, U.K.

**Keywords:** skin penetration, infrared spectroscopy, Raman spectroscopy, stratum corneum sampling, drug uptake into skin

## Abstract

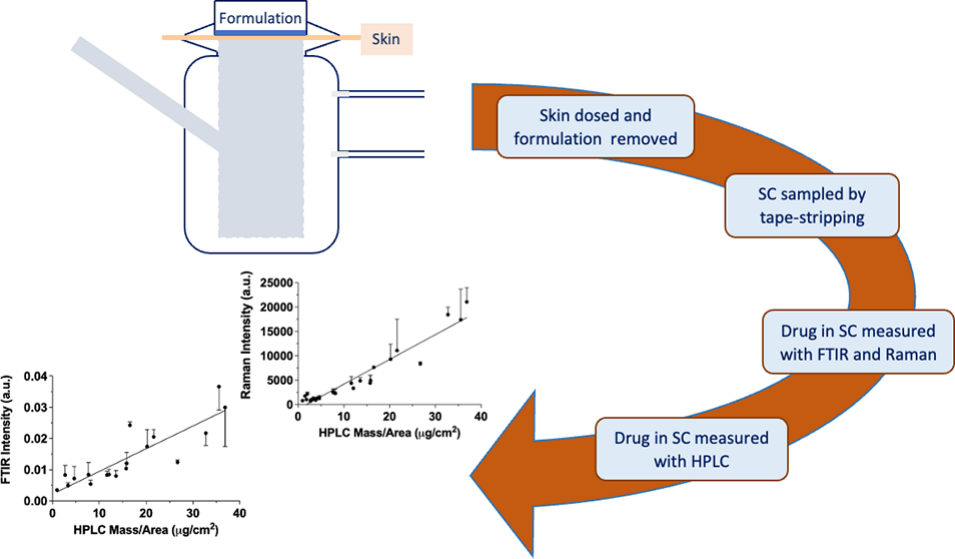

Evaluation of the bioavailability of drugs intended to act within the skin following the application of complex topical products requires the application of multiple experimental tools, which must be quantitative, validated, and, ideally and ultimately, sufficiently minimally invasive to permit use in vivo. The objective here is to show that both infrared (IR) and Raman spectroscopies can assess the uptake of a chemical into the stratum corneum (SC) that correlates directly with its quantification by the adhesive tape-stripping method. Experiments were performed ex vivo using excised porcine skin and measured chemical disposition in the SC as functions of application time and formulation composition. The quantity of chemicals in the SC removed on each tape-strip was determined from the individually measured IR and Raman signal intensities of a specific molecular vibration at a frequency where the skin is spectroscopically silent and by a subsequent conventional extraction and chromatographic analysis. Correlations between the spectroscopic results and the chemical quantification on the tape-strips were good, and the effects of longer application times and the use of different vehicles were clearly delineated by the different measurement techniques. Based on this initial investigation, it is now possible to explore the extent to which the spectroscopic approach (and Raman in particular) may be used to interrogate chemical disposition deeper in the skin and beyond the SC.

## Introduction

Research into methods to assess the bioavailability of the active ingredient from a complex drug product has intensified over the last several years.^1^ This is particularly true for formulations designed to deliver dermatological drugs for which assessment of the rate and extent at which the active compound reaches its site of action, or somewhere near, within the skin itself is required.^2^

While microdialysis and open-flow microperfusion techniques currently offer the closest approach for measuring local concentrations of active compounds that elicit their pharmacological effects in the viable skin layers and can do so in vivo as well,^3,4^ the methods are technically demanding and resource intensive. Other in vivo options, either stratum corneum (SC) sampling (tape-stripping) or “classic” blood level monitoring, despite recent evidence that they have potential value at least with respect to the assessment of cutaneous bioequivalence between products^5−7^ have yet to receive full endorsement by key regulatory bodies.

In contrast, the in vitro release test (IVRT) and the in vitro (skin) penetration test (IVPT) have progressed in terms of broader acceptance and are included in a number of recent product-specific guidances issued by the US Food & Drug Administration.^8,9^ IVRT, of course, is now fully integrated into the quality component of a drug product’s evaluation but does not provide any information pertinent to in vivo safety and efficacy.^10^ IVPT continues to be the workhorse of formulation development and optimization and provides metrics relatable to the rate and extent with which a drug is released from its product and subsequently transports through an excised skin sample.^11,12^ However, because the skin in vitro lacks a functional microcirculation, the IVPT methodology cannot directly yield information on drug levels at or near the site of action within the epidermis/dermis.^13^

The state-of-the-art with respect to the determination of local skin bioavailability and bioequivalence includes stand-alone approaches such as conventional clinical trials and (for glucocorticoids) the vasoconstriction assay. However, no single, specific experimental tool or technique method has yet been adopted for routine application; rather, the current consensus is that a “weight of evidence” process makes sense in that, while all options are deficient to some extent, they do not all suffer from the same flaws.

The work described in this paper represents an initial step in a research program designed to examine the potential of Raman spectroscopy, in particular, to provide an additional component to the toolbox for the assessment of skin bioavailability. Raman and infrared spectroscopies have already been used to track the penetration of chemicals across the SC, both ex vivo and in vivo, in a mostly semiquantitative fashion;^13^ in some cases, the use of nonlinear, stimulated Raman scattering has also allowed the pathways of permeation to be imaged in real-time.^14^ Key advantages of a spectroscopic approach are that measurements can be made noninvasively—no tape-stripping or microdialysis tube insertion in the dermis—and that commercially available instrumentation is available for in vivo studies.^15^

Important ultimate objectives are to determine the extent to which Raman spectroscopy can probe beyond the SC, and how signal attenuation with increasing depth and background interference with signal from the drug can be managed, so as to permit useful metrics pertaining to cutaneous bioavailability (and bioequivalence) to be determined. In the research reported here, the principal goal is to demonstrate that vibrational spectroscopies are able to faithfully report on the uptake of a chemical into the SC when compared to the direct quantification provided using a careful tape-stripping procedure. This initial effort is undertaken ex vivo using porcine skin (an acknowledged and broadly accepted model for the human membrane^16^) and 4-cyanophenol as a model permeant because it possesses a strong Raman signal (−C≡N vibration) in a frequency range where skin and the formulation solvents used are spectroscopically “transparent”,^17^

## Materials and Methods

### Materials

4-Cyanophenol (CP), propylene glycol (PG) and all other solvents and chromatography reagents were obtained from Sigma Aldrich (Dorset, UK). Fresh abdominal porcine skin, originating from a single animal, was obtained from a local abattoir, dermatomed (Zimmer, Hudson, OH, USA) to a nominal thickness of 750 μm, frozen within 24 h of slaughter, and thawed before use. Visible hairs were trimmed carefully with scissors.

### Solubility Determination

The solubility of CP was determined (in triplicate separate measurements) in pure water, in various mixtures of pure water and PG (90:10 to 30:70 v/v), and in PG. The method involved stirring excess quantities of CP with the relevant solvent for 24 h in an oven at 25 °C. Samples were then taken and, after filtration (0.45 μm nylon membrane, SMI-Labhut, Ltd., Maisemore, UK) and appropriate dilution in 40:60 methanol: 0.1% formic acid aqueous solution, CP was quantified by high-performance liquid chromatography with ultraviolet detection (HPLC-UV)—see below.

### Ex Vivo Measurement of CP Uptake into Skin

Two sets of experiments were conducted involving formulations that comprised saturated solutions of CP in different water/PG mixtures (i.e., to maximize the permeant’s thermodynamic activity and flux across the skin). These simple aqueous-based solutions were chosen because of the reasonably good solubility of CP in water;^18^ PG is a commonly used excipient in topical drug products and was included to improve the penetration of CP into the skin^19^ and therefore amplify the spectroscopic signal intensities and facilitate their correlation with chromatographic analyses. The first experiment involved the application of 170 mg mL^–1^ of CP in 50:50 v/v water/PG for 0.5, 1, and 2 h. The second experiment compared three formulations applied for 1 h: (a) 17 mg mL^–1^ of CP in 90:10 v/v water/PG, (b) 170 mg mL^–1^ of CP in 50:50 v/v water/PG, and (c) 380 mg mL^–1^ of CP in PG alone. The compositions of the formulations were chosen, based on the acquired solubility data, to cover a wide range of CP and PG levels. The selected application times reflected earlier observations that the diffusional lag time for CP transport across the stratum corneum is ∼0.5 h and that steady-state transport is achieved, therefore, in 1.0–1.5 h.^18^

In a static, vertical Franz cell,^20^ thermostatted at 32 °C, 300 μL of each solution were applied to the surface of porcine skin (diffusion area of 2.01 cm^2^). The receptor solution was pH 7.4 phosphate-buffered saline (PBS), a common model for the physiological milieu in terms of pH and ionic composition. The donor compartment of the diffusion cell was covered with Parafilm to avoid evaporation of the components of the formulation. After the given application time, the residual formulation was removed using a pipette and the skin was cleaned by wiping with dry tissue. Control experiments involving the application of placebo versions of the same formulations were performed to confirm that no interference, in terms of CP detection, in the spectroscopic and chromatographic analyses was present.

At the end of each experiment, the disposition of CP in the SC was assessed by the sequential removal of this outer skin layer by tape-stripping. Templates (Scotch Tape, 3 M, The Consortium, UK), with a circular internal area of 2.01 cm^2^, were adhered to the skin, and an adhesive tape strip (2.0 cm × 2.5 cm, Scotch Tape, 3 M) was applied to the treated area, pressed firmly down and removed quickly. The procedure was repeated until 20 strips had been taken.

The mass of skin removed on each tape was determined by weight difference (Sartorius model SE2-F, Sartorius AG, Germany), before and after application to the skin. Before weighing, the tapes were discharged of static electricity (R50 discharging bar and ES50 power supply, Eltex Elektrostatik GmbH, Weil am Rhein, Germany). From this mass, and knowing the area of SC on the tape, it was possible to calculate the SC thickness removed (assuming an SC density of 1 g cm^–3^)^21^ and, hence, the corresponding position (or depth) within the barrier. After weighing, tapes were analyzed for CP by FTIR and Raman spectroscopy. Following these analyses, tapes were extracted individually (extraction solvent: 40:60 methanol: 0.1% formic acid aqueous solution) and analyzed for CP by HPLC-UV. All tapes were stored and transferred between analyses in covered trays to minimize dust contamination and contact with other surfaces.

### Analytical Methods

First, once the SC had been sampled, an infrared spectrum was recorded of each tape-strip removed. The tape was placed SC side down onto the internal reflection element (ZnSe crystal having a trapezoidal cut of 45°) of an attenuated total reflection Fourier transform infrared (ATR-FTIR) spectrometer (Perkin Elmer Spectrum 100 FT-IR Spectrometer with a Universal ATR Sampling Accessory, Norwalk, CT, USA). To ensure reproducible contact between the sample and the crystal, the same pressure was always applied to the tape-strips (force gauge 100 N). The ATR-FTIR spectra were obtained in the frequency range of 4000–650 cm^–1^ with a spectral resolution of 8 cm^–1^ (data interval 2 cm^–1^). The peak positions were assigned using Perkin Elmer Spectrum Version 6.0.2. Integrated signals from the C≡N (2250 and 2200 cm^–1^) stretching vibrations were recorded. Spectra from 3 different places on the same tape were recorded; the area in contact with the ATR element for each replicate was about 3.14 mm^2^. Between successive measurements of a series of tape-strips, any residues on the ZnSe internal reflection crystal were removed with an alcohol wipe.

Second, after the ATR-FTIR measurements, Raman spectra (Renishaw RM1000 Raman microscope and v1.2 WIRE software, Renishaw plc, Wotton-Under-Edge, UK) were acquired from the same tape-strips. A 1200-line/mm grating providing spectral resolution of 1 cm^–1^ was used with a diode laser operating at 785 nm. The Raman band (520 cm^–1^) of a silicon wafer was used for calibration. An exposure time of 10 s with 4 accumulations and a laser power of 100% were used with a long, 50× working objective. All spectra were acquired over the frequency range of 2450–1500 cm^–1^. For each tape, 3 spectra at 3 different places were again acquired and the signals from the C≡N stretching vibration were recorded; in this case, the area examined for each replicate was 2.8 μm^2^. Additional details of the protocol followed to extract the maximum integrated intensities from the two sets of spectroscopic measurements are provided in the Supplementary Information (and illustrated in Figures S1–S3).

Third, the same tape-strips were then extracted individually and analyzed for CP by HPLC-UV (Dionex, Camberley, UK) using Chromeleon software (version 6.80 SP1). A HiQSil C18HS analytical reverse phase column (150 × 4.6 mm i.d.; 5 μm particle size, Kya Technologies Corporation, Japan) was used. The mobile phase was 40:60 methanol:0.1% formic acid aqueous solution. The flow rate was 1.5 mL min^–1^. The injection volume was 50 μL and the retention time of CP was ∼5.3 min. Selectivity of the analytical method was confirmed by extraction of tapes with SC that had not been exposed to CP; no interference was found at the relevant retention time. The limits of detection and quantitation of the HPLC assay were 0.04 and 0.13 μg mL^–1^, respectively. Recovery of CP from tapes^5,7^ was assessed by spiking tape-stripped samples of untreated SC with known amounts (spanning the range found on the tapes when the test formulations were applied) of CP in solution (in 40:60 MeOH:0.1% formic acid) which was left to dry for several hours before extraction. Mean extraction efficiencies were 95 (±2)% from tape-strips and 89 (±4)% from the remaining tissue.^22^

### Data Analysis

The critical level for peak detection (*A*_c_) for the FTIR and Raman data were defined in each individual spectrum by^5,7^
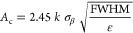
where *k* is the coverage factor to achieve the desired level of confidence, σ_β_ is the standard deviation of the spectral intensity in the background region, FWHM is the full-width at half maximum of the peak, and ε is the step size in the spectrum (and equal to 2 and 0.88 cm^–1^ in the FTIR and Raman experiments, respectively). For peaks whose position is certain (i.e., the peak appears in frequencies within the 6 × FWHM range), an error of the first kind (deciding a peak is present when it is not) will occur with a probability of 5% when *k* is 1.645; σ_β_ is the average standard deviation of the baseline data in the 3FWHM/ε range to the left (2200 cm^–1^ and below) and right (2250 cm^–1^ and above) of the CP peak as illustrated in the Supplementary Information (Figure S4). Any measurements falling below *A*_c_ were considered to be zero.

The integrated CP signals from the two spectroscopic measurements and the extracted quantities of the chemical from the tape strips provide relative and absolute amounts, respectively, per unit area of skin. To display the data as concentration profiles across the SC, the measured values were divided by the corresponding thickness of SC removed on the respective tape-strips and plotted at the mid-point of the cumulative SC depth. The relative SC uptake per unit area of the chemical was calculated by summing the spectroscopic signal (FTIR and Raman) and total CP mass per area (HPLC) from all tape strips. These values, of course, are equivalent, respectively, to the areas under the relative (FTIR and Raman) and absolute concentration (HPLC-UV) profiles plotted as a function of SC depth. Correlations between the relative/absolute sets of concentration data were performed using GraphPad Prism (version 9.3.1, San Diego, CA). When at least one measurement of the three spectroscopic replicates of SC uptake was below the critical level for peak detection, these data were excluded from the correlations. Unless stated otherwise, results are reported as the mean ± standard deviation.

## Results

### Solubility Determination

**Figure 1:**
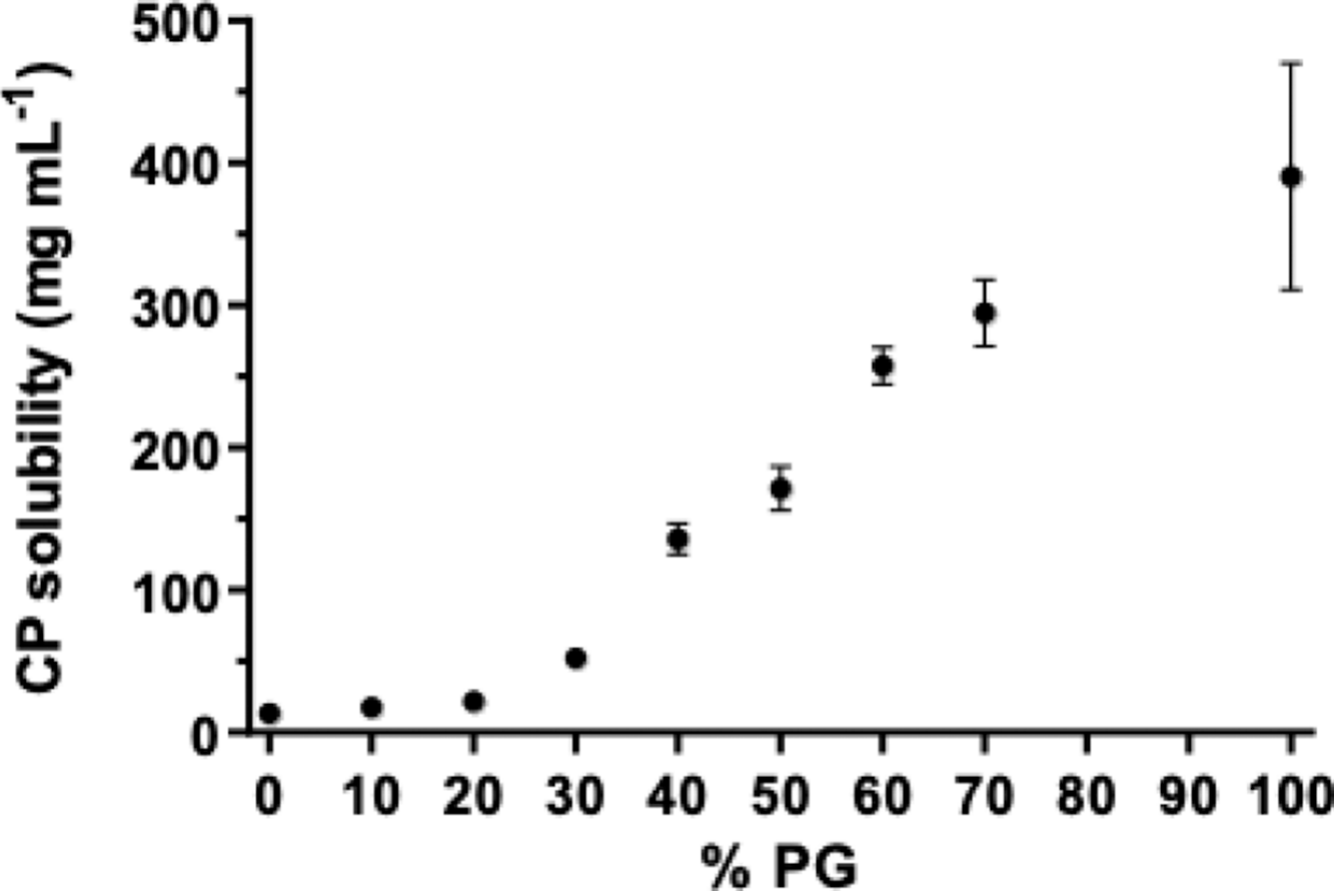
Solubility of 4-cyanophenol (mean ± standard deviation (SD), *n* = 3) in water, in various mixtures of water and PG (90:10 to 30:70 v/v), and in PG.

CP solubility increased with the introduction of PG, from a lower level in water alone (13.4 ± 2.1 mg mL^–1^), to a value that was nearly 30-fold higher in the pure cosolvent (391 ± 80 mg mL^–1^) (Figure 1).

### CP Uptake into Skin as a Function of Time

**Figure 2:**
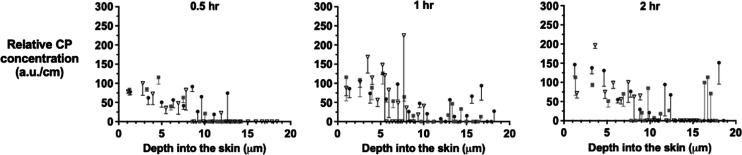
FTIR-assessed CP relative concentration (i.e., the maximum signal intensity of the C≡N vibration at 2230 cm^–1^ divided by the SC thickness removed on successive tape-strips) as a function of the mid-point of the SC depth interval collected following the application of a 170 mg mL^–1^ solution of the chemical in 50:50 v/v water/PG for 0.5, 1, and 2 h. Data points are the means of three measurements taken from each tape strip minus SD. Tape strips from the three replicates are designated by different symbols.

**Figure 3:**
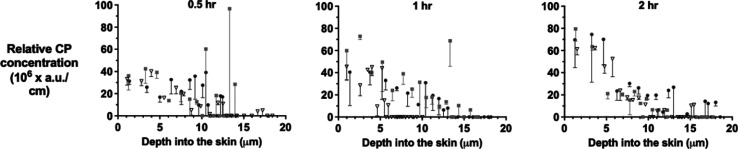
Raman-assessed CP relative concentration (i.e., the maximum signal intensity of the C≡N vibration at 2230 cm^–1^ divided by the SC thickness removed on successive tape-strips) as a function of the mid-point of the SC depth interval collected following application of a 170 mg mL^–1^ solution of the chemical in 50:50 v/v water/PG for 0.5, 1, and 2 h. Data points are the means of three measurements taken from each tape strip minus SD. Tape strips from the three replicates are designated by different symbols.

**Figure 4:**
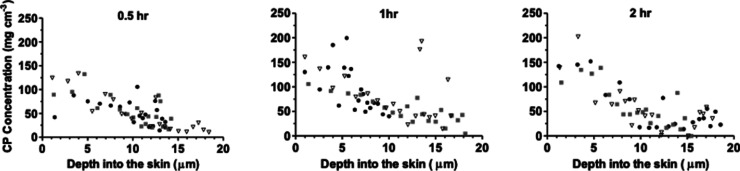
HPLC-UV assessed CP concentration (in μg cm^–3^) as a function of the mid-point of the SC depth interval collected following application of a 170 mg mL^–1^ solution of the chemical in 50:50 v/v water/PG for 0.5, 1, and 2 h. Data points represent the experimentally extracted amounts of CP divided by SC thickness on each tape. Tape strips from the three replicates are designated by different symbols.

The ATR-FTIR analyses of the SC on the tape-strips following the application of 170 mg mL^–1^ of CP in 50:50 v/v water/PG for 0.5, 1, and 2 h are presented in Figure 2 for three replicate experiments. Each panel shows the profile of the C≡N signal divided by the SC thickness on the corresponding tape-strip as a function of position across the SC—in other words, a relative CP concentration versus SC depth profile. The Raman spectroscopic results acquired from the same strips are presented in the same way in Figure 3. Finally, CP was extracted from the spectroscopically analyzed tape-strips and quantified by HPLC-UV. The resulting CP concentration on each tape strip, calculated by dividing the CP mass per unit area by the thickness of the SC on that tape strip, is presented as a function of time and depth in Figure 4.

The sums of the signal intensity (for the FTIR and Raman data) or the mass of drug (for the HPLC-UV results) of all the tape strips from an SC sample (i.e., the total response) are summarized in Table 1 and Figure S5 in the Supplementary Information. The trends observed by each of the three assessment methods are consistent and, from the evolution of CP uptake over time, the diffusional lag time (*t*_lag_) can be deduced (as explained in the Supplementary Information). The *t*_lag_ estimated from the HPLC data is 0.68 h, a value comparable to that reported previously for CP uptake into human skin in vivo from a saturated aqueous solution (0.54 ± 0.10 h).^18^ Estimated *t*_lag_ values derived from the FTIR and Raman data are similar although slightly larger (0.91 and 1.06 h, respectively).

**Table 1:** Total Spectroscopic Signal (FTIR and Raman) and Total CP Mass per Area (HPLC) from All Tape Strips Following Application of One Saturated Formulation (170 mg mL^–1^ of CP in 50:50 v/v Water) for Three Different Application Times^a^

application time (h)	total response (mean ± SD)		
	10^–2^ × FTIR (a.u.) [*n* = 3]	10^3^ × Raman (a.u.) [*n* = 3]	HPLC (μg cm^–2^) [*n* = 3]
0.5	6.2 ± 0.3	32 ± 4.5	96 ± 17
1.0	7.9 ± 0.8	37 ± 16	119 ± 20
2.0	9.6 ± 1.9	48 ± 8.0	126 ± 13

a Spectroscopic results represent the mean ± SD of the three SC samples calculated from the summation of the mean value of 3 measurements as a function of depth for each SC sample; HPLC-UV data represent the mean ± SD of single measurements of the three SC samples.

### CP Uptake into Skin from Different Solvents

**Figure 5:**
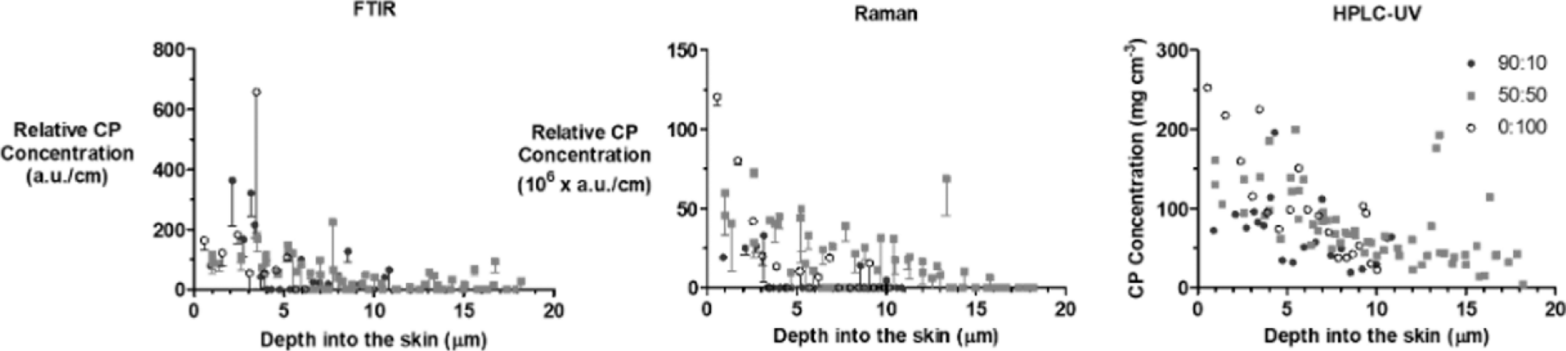
CP concentration versus SC depth profiles assessed (a) by FTIR (as the maximum signal intensity of the C≡N vibration), (b) by Raman spectroscopy (again as the C≡N signal), and (c) by HPLC-UV, all divided by the SC thickness, following the application of three formulations of the chemical for 1 h. Data points for the spectroscopy measurements are the means minus SDs of three measurements on each tape strip taken from one piece of skin (90:10 and 0:100 water/PG v/v formulations) or from three pieces of skin (50:50 v/v formulation). For the HPLC-UV assessment, data points represent the experimentally determined concentrations of CP extracted from the individual tapes from either one (90:10 and 0:100 v/v) or three (50:50 v/v) pieces of skin.

Figure 5 shows the concentration profiles of CP in the SC, assessed by FTIR, Raman, and HPLC-UV, after a 1 h of application of three saturated formulations: (a) 17 mg mL^–1^ of CP in 90:10 v/v water/PG, (b) 170 mg mL^–1^ of CP in 50:50 v/v water/PG, and (c) 380 mg mL^–1^ of CP in PG alone. The total spectroscopic response (FTIR and Raman) and total CP mass per area (HPLC) from all tape strips provide measures of the relative uptakes of the chemical (Table 2) and these values—as before—are equivalent to areas under the relative (FTIR and Raman) and absolute concentration (HPLC-UV) profiles plotted as a function of SC depth.

**Table 2:** Total Spectroscopic Signal (FTIR and Raman) or Total CP Mass per Area (HPLC) from All Tape Strips Following Application of Three Different Formulations for 1 h Measured in a Single Experiment for the 90:10 and the 0:100 Formulations and in Triplicate for the 50:50 Formulation (Table 1)^a^

formulation (H_2_O/PG %v/v)	total response (mean ± standard deviation (SD))		
	10^–2^ × FTIR (a.u.)	10^3^ × Raman (a.u.)	HPLC (μg cm^–2^)
90:10	8.6 ± 0.3 [3]	8.7 ± 0.3 [3]	64 [1]
50:50	7.9 ± 0.3 [3]	37.0 ± 1.1 [3]	119 ± 20 [3]
0:100	7.5 ± 0.4 [3]	28.5 ± 0.3 [3]	121 [1]

a FTIR and Raman results for the 90:10 and 0:100 formulations are the sum of the mean value of 3 sites on each tape strip ± the SD of the 3 sites measured on each tape strip pooled across all tape strips. FTIR and Raman results for the 50:50 formulations are the sum of the mean value of 3 sites on each tape strip averaged across 3 pieces of skin ± the pooled SD of the three sites measured on each tape strip averaged across the three pieces of skin. The HPLC results are the total CP mass per area on all the tape strips for the 90:10 and 0:100 formulation and the average of the total CP mass per area on all the tape strips averaged across three pieces of skin for the 50:50 formulation. The SD for the HPLC results for the 50:50 formulation is that of the total CP mass in three pieces of skin. Note that the spectroscopy SDs, which quantify the variability of the 3 measurements on each tape strip, differ from the HPLC SD, which quantifies the variability of the measurements between skin pieces (i.e., these SD values cannot be used in statistical analyses comparing HPLC results with those from FTIR and Raman).

## Discussion

As expected, the solubility of CP was significantly impacted by the relative amount of water to PG in which the chemical was dissolved (Figure 1). The 50:50 mixture was chosen as a median between the solubilities in the pure solvents and used to investigate the chemical’s disposition in the SC as a function of time. To examine the potential effect of PG on the uptake of CP into the skin, the 50:50 formulation was then compared with one comprising water/PG in the ratio of 90:10 and to another in pure PG alone with CP at its solubility limit in each.

Figures 2 and 3 show the relative CP concentration profiles determined by FTIR and Raman spectroscopy, respectively, across the SC after 0.5, 1, and 2 h applications of a saturated solution of the chemical in 50:50 v/v water/PG. While the measured maximum signal intensities in arbitrary units are different for the two approaches used (i.e., absorption versus scattering of the incident radiation for FTIR and Raman, respectively), the results are consistent with previously published absolute concentration profiles^18,23,24^ and with those in Figure 4; specifically, the spectroscopic data also show an increasing uptake of CP with time of application (consistent with the correspondingly larger areas under the profiles reported in Table 1) and decreasing CP concentration profiles with increasing depth.

**Figure 6:**
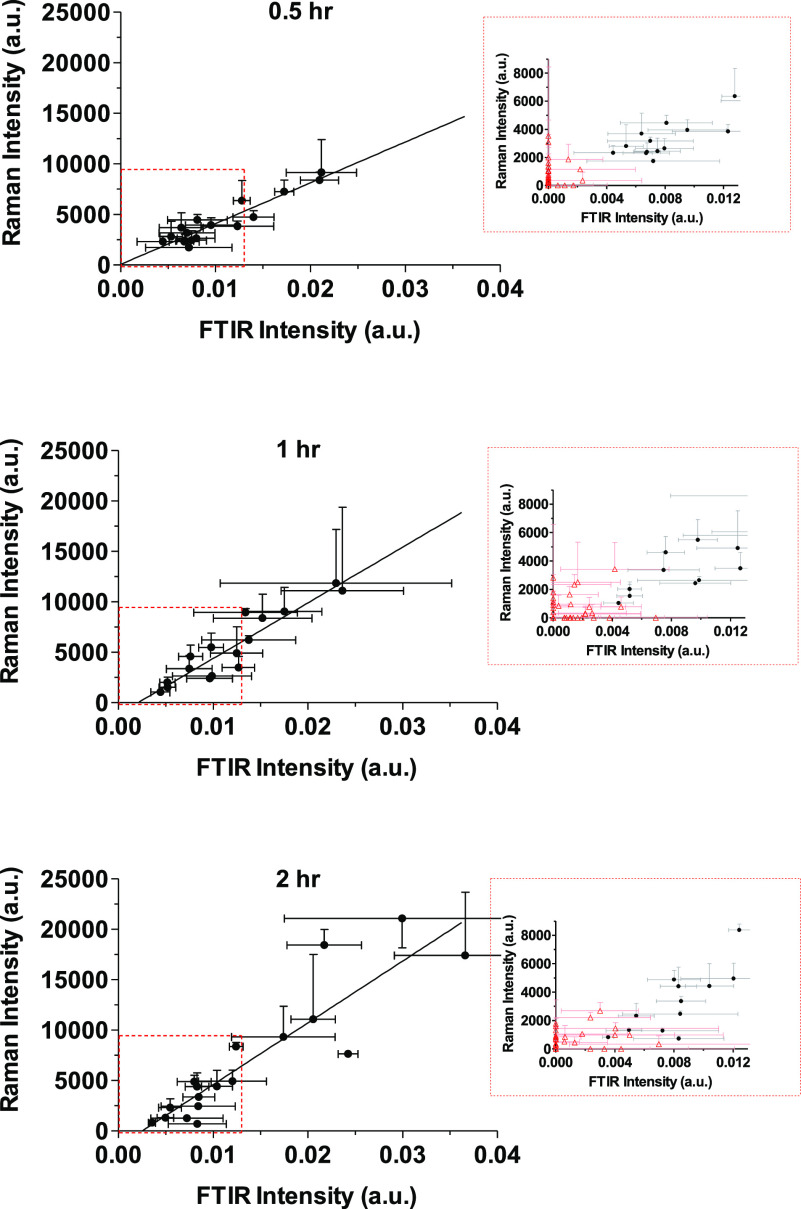
Correlations between the relative CP signal intensities acquired with FTIR and Raman spectroscopies (mean plus (or minus when required to facilitate visualization) SD of 3 measurements for each tape strip); the line represents the linear regression gradient. Expanded views of the data closest to the origin are also shown; when at least one of the triplicate measurements was below the critical level for peak detection (shown as red triangles), these data were excluded from the correlations.

Correlations between the Raman and FTIR results at each of the three application times are presented in Figure 6. Linear regressions through the Raman versus FTIR data yielded gradients—in dimensionless units—of 4.0 × 10^5^, 5.5 × 10^5^, and 6.1 × 10^5^, at 0.5, 1, and 2 h, respectively; the corresponding *r*^2^ values were 0.88, 0.86, and 0.81. The 95% confidence intervals of the gradients were (3.2–4.9) × 10^5^ at 0.5 h, (4.2–6.8) × 10^5^ at 1 h, and (4.6–7.7) × 10^5^ at 2 h. While the gradients increased by less than 1.5-fold over time, there was a significant difference (*p* < 0.05) between those at 0.5 and 2 h. This is due to the slope being influenced by the higher values measured in the first tapes of the 1 and 2 h uptake experiments. These initial tape strips may be most susceptible to variation in the amounts of SC collected from the three randomly selected sites (as compared to the HPLC-UV measurements which integrate across the entire tape strip surface area). Of course, variability was also observed in the data acquired from tape-strips at the deeper layers of the SC where the levels of CP detected were the lowest of those measured and closest to the critical level of detection of the two spectroscopic measurements employed.

**Figure 7:**
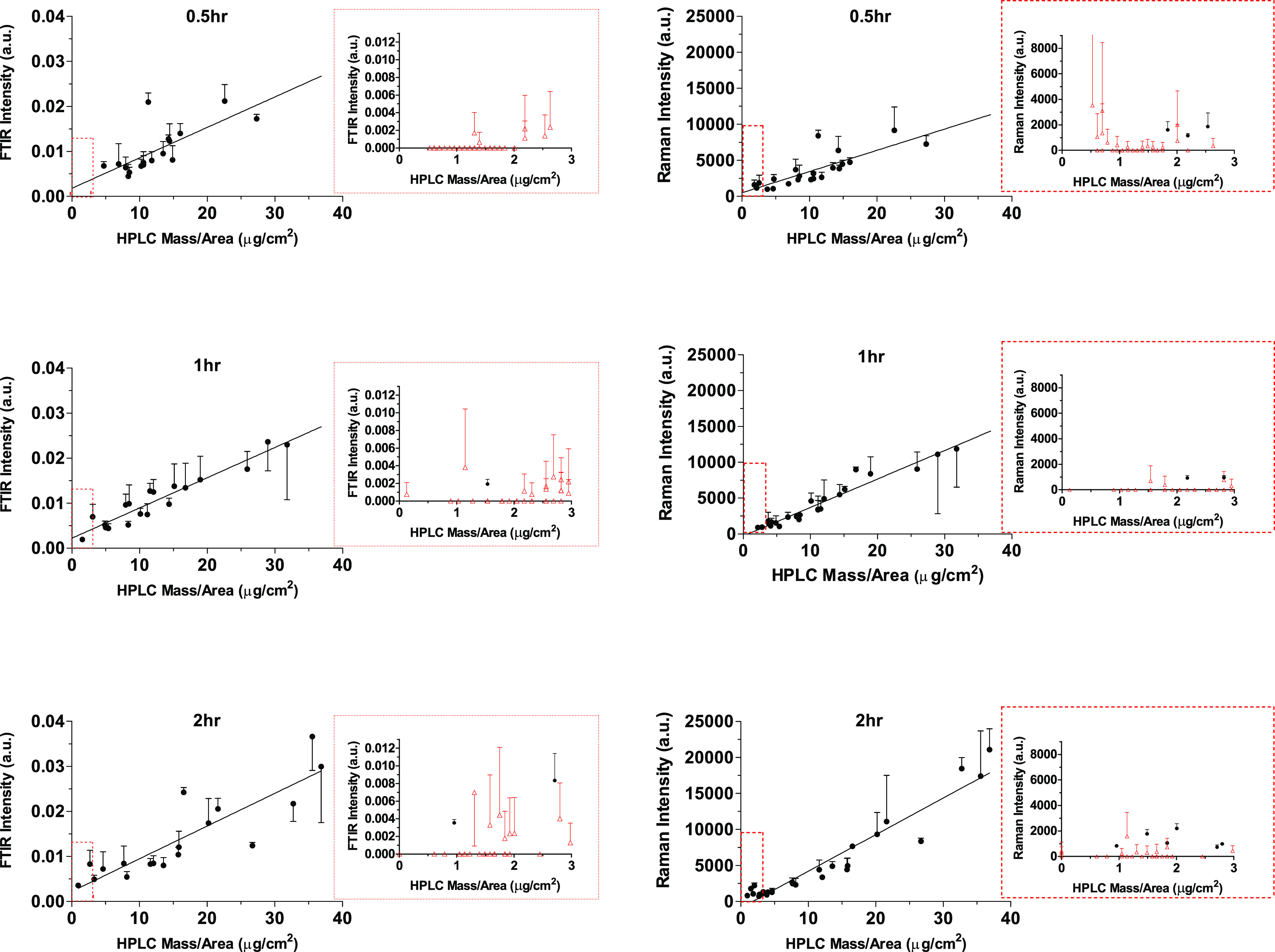
Correlations, as a function of time, between the relative CP intensities determined from the maximum C≡N vibration signal, acquired with FTIR and Raman spectroscopies (left and right panels, respectively), with the corresponding amount per area of the chemical extracted from the SC on the corresponding tape strips; spectroscopic results presented as mean plus (or minus when required to facilitate visualization) SD of three measurements on each tape strip. Expanded views of the data closest to the origin are also shown; when at least one of the triplicate measurements was below the critical level for peak detection (shown as red triangles), these data were excluded from the correlations.

Importantly, both sets of spectroscopic measurements correlated well with the CP concentration in the corresponding tape strips. The correlations between the datasets at 0.5, 1, and 2 h are shown in Figure 7 and details of the linear regressions are summarized in Table 3. The gradients of the FTIR versus HPLC datasets are not affected by treatment time whereas those for the Raman-HPLC were significantly different (*p* < 0.05), albeit by less than a factor of 1.7, consistent with the gradient increases observed in the linear regressions of Raman versus FTIR results (Figure 6). The ratios of the Raman and FTIR versus HPLC gradients are, as expected, similar to the gradients of the Raman versus FTIR data sets (i.e., 4.3 × 10^5^, 5.9 × 10^5^, and 7.0 × 10^5^ for treatment times of 0.5, 1, and 2 h, respectively. Sensitivity to the higher values measured in the first tapes as well as variability in the data acquired from tape-strips at the deeper layers are, as in Figure 6, contributors to these differences. An additional factor may be the different depths into the SC samples “interrogated” by the two spectroscopic techniques; while FTIR in the reflectance mode typically penetrates to a few tenths of a micron, the Raman signals indicate complete penetration through the SC (as evidenced by measurable signals originating from the tape adhesive in all spectra acquired). The different axial resolutions of the two techniques could also explain the higher Raman intensities (Figure 6) for which the information is collected from all the stratum corneum collected on each tape. This would also explain the better sensitivity of Raman to detect the greater CP levels present in the first tapes. Nonetheless, the spectroscopic analyses of CP in the SC are able to provide a faithful representation of the applied chemical’s relative disposition that is sensitive to the experimental variable interrogated in this part of the study, namely the duration of contact with the formulation.

**Table 3:** Gradients of the Linear Regressions of the Datasets Presented in Figure 7^a^

	FTIR C≡N vs CP amount			Raman C≡N vs CP amount		
time (h)	0.5	1.0	2.0	0.5	1.0	2.0
gradient (a.u. μg^–1^ cm^2^)	6.8 × 10^–4^	6.7 × 10^–4^	7.3 × 10^–4^	294	397	508
95% C.I. (a.u. μg^–1^ cm^2^)	3.2 × 10^–4^, 10.3 × 10^–4^	5.6 × 10^–4^, 7.8 × 10^–4^	5.1 × 10^–4^, 9.4 × 10^–4^	195, 394	354, 440	447, 569
*r*^2^	0.52	0.91	0.76	0.66	0.94	0.92

a The FTIR versus CP amount gradients are not significantly different from one another, whereas those for Raman are significantly different to each other (*p* < 0.05).

Finally, the results in Figure 5 and Table 2 provide further confirmation that the spectroscopy methods employed in this work can track and, in the case of Raman, differentiate the disposition of a chemical when delivered from different formulations. The FTIR and Raman assessments lined up in a relatively close correlation (which could be improved by increasing the number of replicates) with the chemical’s uptake profile within the SC determined quantitatively on the sequentially collected tape-strips. Because the three vehicles each contained CP at saturation (meaning they all have the same thermodynamic driving force) they should, in the absence of alteration of the SC by one or more of the excipients used, have delivered the compound into the skin with the same efficiency.^25,26^ However, it appears from the preliminary results in Table 2 that the relative amounts of water and PG in a formulation may exert a noticeable effect on the disposition of CP as has been identified before.^19,27,28^ Specifically, from the Raman and HPLC measurements, CP uptake into the SC was smaller from the 10% PG formulation compared with the 50 and 100% PG formulations, which were similar to each other. The reduced CP uptake from the 10% PG formulation is less clear from the FTIR data, perhaps in part because the FTIR measurements are noisier than those from the Raman. Definitive conclusions on the PG effect will only be possible once additional experiments have been performed.

**Figure 8:**
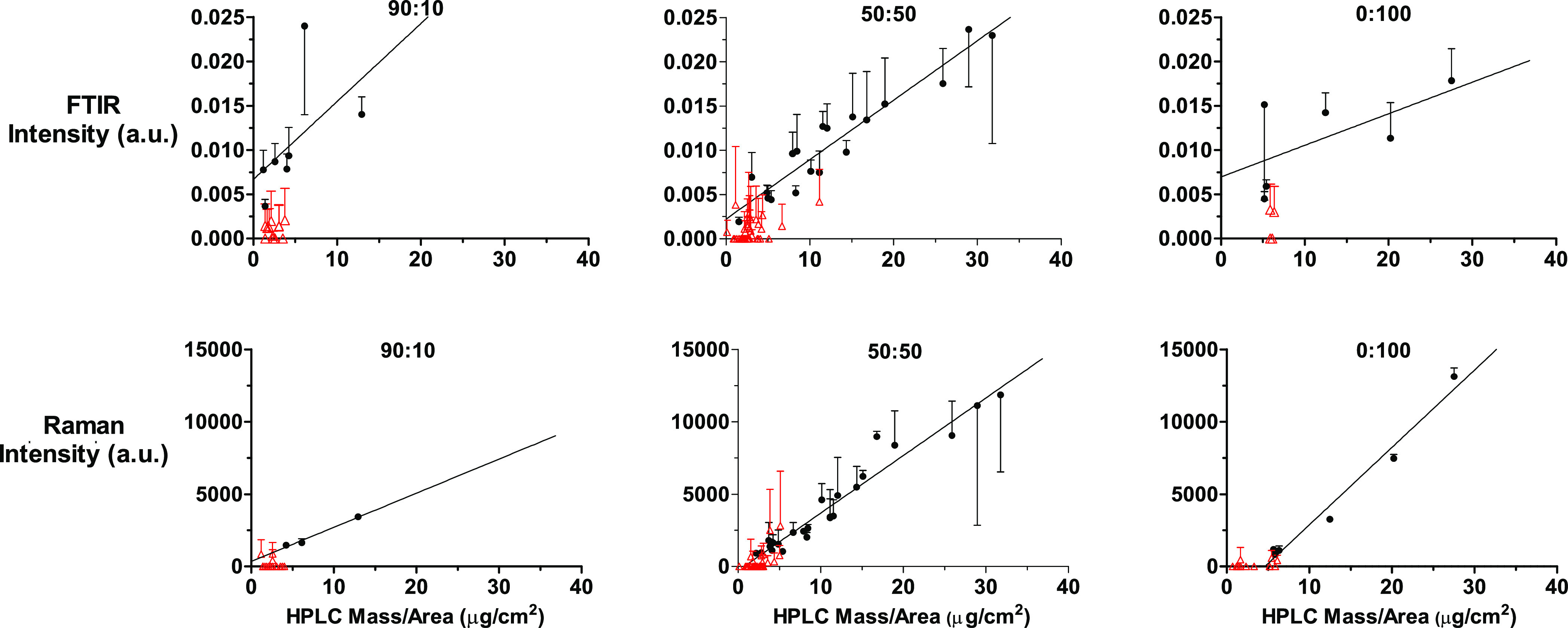
Correlations, as a function of formulation applied for 1 h, between the relative CP intensities determined from the maximum C≡N vibration signal, acquired with (upper panels) FTIR and (lower panels) Raman spectroscopies, with the corresponding amount per area of the chemical extracted from the SC on the corresponding tape strips; spectroscopic results presented as mean plus or minus SD of three measurements on each tape strip. Data with at least one measurement below the critical level for peak detection are shown as red triangles and were not included in correlations.

The relationships of the FTIR and Raman data with the corresponding CP masses per area in the tape-stripped SC for the three formulations are presented in Figure 8 with the linear regressions shown being summarized in Table 4. It appears that the spectroscopic response is smaller for the 10% PG formulation than for either the 50% PG or the pure PG vehicles, which are similar. This observation, if confirmed in replicate studies, could indicate that increasing the amount of PG in the SC above that amount in the 90:10 formulation may increase the intensity of both the FTIR and Raman measurements. Alternatively, the apparent difference in the 10% PG formulation may arise because this formulation delivers less CP to the SC (and, indeed, it is the case that more of the data are closer to the limit of the detection, consistent with the poor correlation coefficient seen for 90:10 formulation data (Table 4)). That having been said, it is possible to rule out—by a simple ‘back-of-the-envelope calculation presented in the Supplementary Information—that depletion of CP from the 90:10 formulation (in which the chemical is present at the lowest concentration) is unlikely to be the cause of the reduced uptake of CP into the SC.

**Table 4:** Gradients of the Linear Regressions of the Datasets Presented in Figure 8^a^

	FTIR C≡N vs CP amount			Raman C≡N vs CP amount		
formulation (%v/v water/PG)	90:10	50:50	0:100	90:10	50:50	0:100
gradient (a.u. μg^–1^ cm^2^)	8.8 × 10^–4^	6.7 × 10^–4^	3.6 × 10^–4^	235	397	534
95% C.I. (a.u. μg^–1^ cm^2^)	–6.9 × 10^–4^, 24.5 × 10^–4^	5.6 × 10^–4^, 7.8 × 10^–4^	–2.5 × 10^–4^, 9.6 × 10^–4^	–142, 613	354, 440	419, 650
*r*^2^	0.29	0.91	0.40	0.98	0.94	0.98

a There are significant differences between the 50:50 and 0:100 gradients of the FTIR versus CP amount (*p* < 0.05) and between the Raman versus CP amount gradients for the 90:10 and 0:100 (*p* < 0.0001) and 50:50 and 0:100 (*p* < 0.05) formulations.

## Conclusions

The research presented above describes the acquisition of information pertinent to the assessment of topical drug bioavailability and demonstrates a high degree of correlation between the established method of SC sampling (tape-stripping) and the application of both FTIR and Raman spectroscopies. While the latter approaches are semiquantitative, they are able to assess the relative performance of different formulations—an attribute essential when addressing the question of bioequivalence—and they do so without the laborious steps of extracting the chemical from the tape-strips and its subsequent chromatographic analysis. Of the two spectroscopies, FTIR in the reflectance mode has been employed for many years to probe the SC specifically but has not permitted interrogation of the living skin layers beyond. Raman, in the confocal mode, on the other hand, has the potential to “see” deeper into the skin in areas closer to the sites of action of the majority of drugs used in dermatology. To pursue this potential application further will require that two particular challenges are addressed: (a) to ensure that the Raman signal of the chemical of interest is measurable and distinguishable from the background spectrum from the skin and the excipients used and (b) to permit signal attenuation to be assessed with increasing depth into the skin so that an appropriate and validated correction can be applied.

## Supporting Information Available

(a) The approach used to analyze the acquired Raman spectra, specifically the baseline correction method and the quantification of the signal via the center, width, height, %gaussian, and area of the peak; (b) determination of the critical level for peak detection by application of the approach described in ISO 19668; and (c) mathematical analysis of 4-cyanophenol uptake into skin as a function of time—comparison of theory versus experiment and determination of a diffusional lag-time (PDF)
