# Supplementary material for: Quantification of Chemical Uptake into the Skin by Vibrational Spectroscopies and Stratum Corneum Sampling
Source: Mol Pharm. 2023 Apr 13;20(5):2527–35. doi: 10.1021/acs.molpharmaceut.2c01109 (PMC10155209; doi:10.1021/acs.molpharmaceut.2c01109)
Supplement: Supplementary file 1 — mp2c01109_si_001.pdf [file mp2c01109_si_001.pdf]

Supplementary Information

Quantification of chemical uptake into the skin by vibrational spectroscopies and stratum corneum sampling

M. Alice Maciel Tabosa[1]*, Pauline Vitry[1]*, Panagiota Zarmpi[1], Annette L. Bunge[2], Natalie A. Belsey[3], Dimitrios Tsikritsis[3], Timothy J. Woodman[1], K.A. Jane White[4], M. Begoña Delgado-Charro[1], and Richard H. Guy[1‡]

Raman spectra analysis

Figure S1 shows an example of a confocal Raman spectrum collected from a tape-strip on which a sample of stratum corneum (SC) that had been treated with a saturated solution of 4-cyanophenol (CP) is present. The peaks at 1600 and 2230 cm^{-1} originate from CP, corresponding to the aromatic C=C vibration and the intense vibrational frequency of the C≡N bond, respectively. The signal at ~1740 cm^{-1} comes from a component of the tape and a very small absorbance centred at about 1650 cm^{-1} is the amide I vibration from (primarily) keratin in the SC.

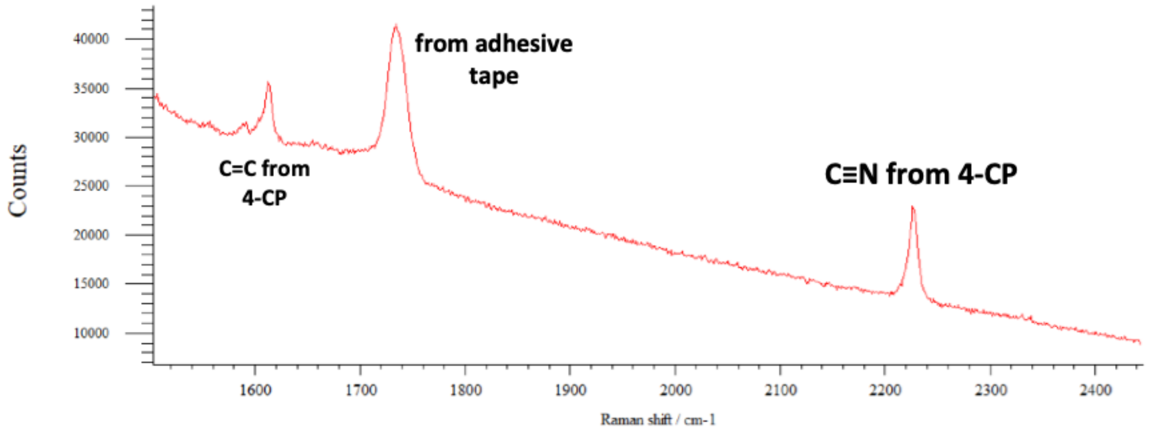

Figure S1: Typical Raman spectrum acquired from SC removed on a tape-strip.

To enable an accurate assessment of the maximum C≡N signal intensity, it is first necessary to correct for the non-constant baseline and this is accomplished using the "intelligent" polynomial fitting available in the Wire 4.4 software package (Renishaw, Gloucestershire, U.K.), producing the result shown in Figure 2.

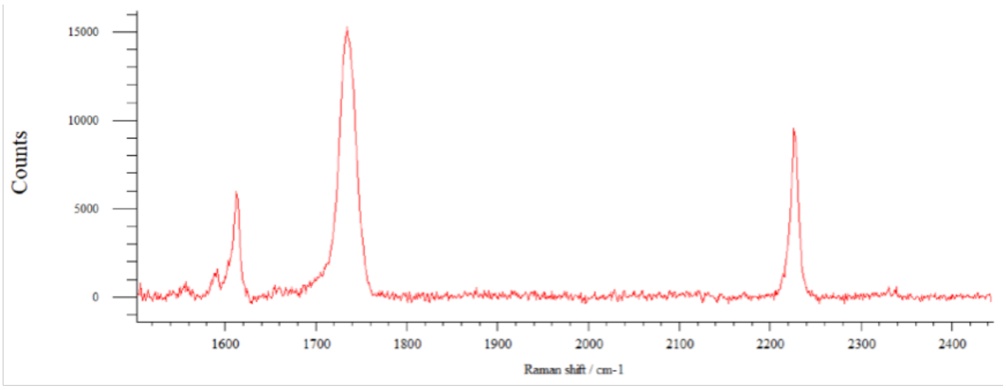

Figure S2: The baseline-corrected Raman spectrum shown in Figure S1.

Subsequently, the curve fitting tool is used to assess the CP signal peak of interest at 2230 cm^{-1}. This procedure enables the centre, width, height, %gaussian and area of the peak to be determined as shown in Figure S3. The peak height is recorded as the maximum CP intensity.

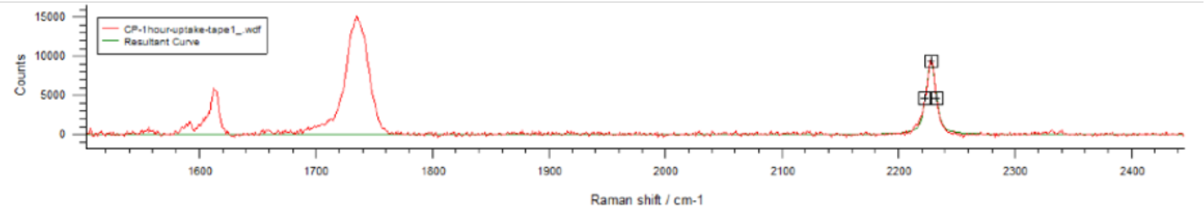

Curve Na...	Centre	Width	Height	% Gaussi...	Type	Area	ChiSq
Curve 1	2228.51	9.875	9421.07	41.7489	Mixed	126470	144.881

<u>Figure S3</u>: Results of fitting the peak of interest in the baseline-corrected Raman spectrum shown in Figure S2.

Determination of the critical level for peak detection

Application of the approach described in ISO 19668[22] is illustrated in Figure S4 below.

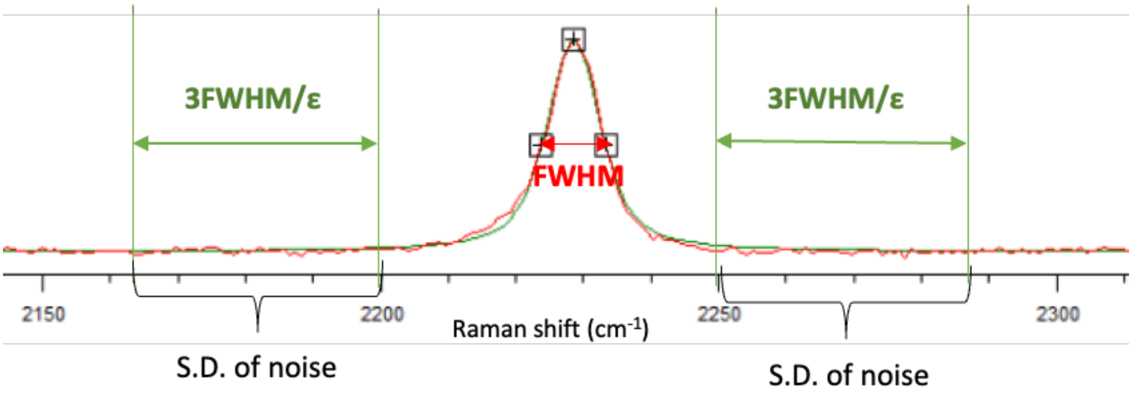

<u>Figure S4</u>: The critical level for detection of a peak (A_C) is defined by: $A_c = k \times \sigma_\beta \times (6 \times FHWM/\varepsilon)$ [terms defined in the main text]. If the coverage factor (k) is 1.645, then the probability that a peak is present, when it actually is not, is 5% (i.e., there is a 95% probability that the peak is real).

A more conventional assessment of the signal-to-noise ratio (SNR) was also performed on the data using SNR = 5 in accord with the document: *"Bioanalytical Method Validation – Guidance for Industry"* (U.S. Department of Health and Human Services, Food and Drug Administration, Center for Drug Evaluation and Research (CDER), Center for Veterinary Medicine (CVM), May 2018, Biopharmaceutics). The measurements identified as falling below the relevant value of A_C overlapped very closely with those determined to have SNR < 5.

4-Cyanophenol uptake into skin as a function of time

The total spectroscopic response (FTIR and Raman) and total CP mass per area (HPLC) from all tape increases over time (t) relative to the lag time (t_{lag}) as predicted by the following equation:

$$\frac{M_{SC}}{C_{SC,0}AL_{SC}}=\frac{1}{2}-\frac{4}{\pi^2}\sum_{n=0}^{\infty}\frac{1}{(2n+1)^2}\exp\left[-\frac{(2n+1)^2\pi^2 t}{6t_{lag}}\right]$$

derived from a one-dimensional diffusion model describing chemical uptake (M_{sc}/A) into an initially drug free membrane of thickness L_{sc} following the application of a constant concentration source ($C_{sc,0}$); values of t_{lag} can be derived by fitting the above equation to the data in Table 1 of the paper (see Figure S5 below).

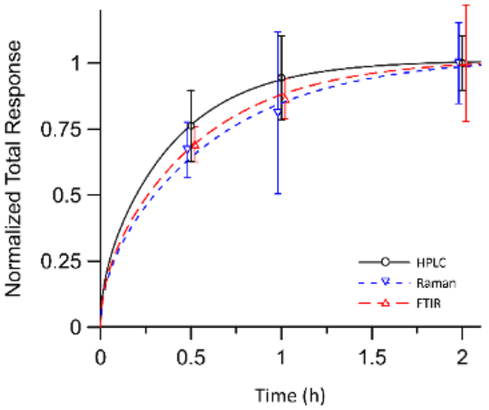

<u>Figure S5</u>: Total spectroscopic signal (FTIR and Raman) and total CP mass per area (HPLC) from all tape strips following application of one saturated formulation (170 mg mL^{-1} of CP in 50:50 v/v water/propylene glycol) for three different application times normalized by the result at 2 h. To facilitate viewing, the FTIR and Raman data are offset by ±1.5 minutes from the actual measurement time. Curves represent the best-fit regressions to the cumulative uptake calculated from a pseudo-homogenous one-dimensional diffusion model with a constant concentration source on the surface of an initially drug free membrane for a lag time of 0.68 h (HPLC), 1.06 h (Raman), and 0.91 h (FTIR).

4-Cyanophenol depletion from the 90:10 water-propylene glycol formulation?

The calculation uses published steady-state CP flux (J_{ss}) measurements across human skin[a,b] (~100 µg cm^{-2} h^{-1}), and an estimate of the steady-state mass per area in the SC ($M_{SC,ss}/A$) from the 50:50 and 0:100 water:PG solutions (~130 µg cm^{-2} at 1 h and approximately the same as that measured from the 50:50 solution at 2 h), in the following equation (derived from Eq. 20 in an earlier publication[c] assuming that the ratio of the SC to viable epidermal permeabilities is large) to determine the amount per unit area (M_{in}/A) that enters the SC in a time t:

$$M_{in}/A = (J_{ss} \text{ x } t) + (2M_{SC,ss}/3A) = (100\ \mu\text{g cm}^{-2}\ \text{h}^{-1} \text{ x } 1\ \text{h}) + ((2 \text{ x } 130\ \mu\text{g cm}^{-2})/3) = 187\ \mu\text{g cm}^{-2}$$

The cumulative mass that might have absorbed in 1 hour is therefore less than 10% of the amount of CP applied (0.3 mL containing 17 mg mL^{-1} of CP on 2 cm^2 corresponds to 2.55 mg cm^{-2}).

a Pirot, F.; Kalia, Y. N.; Stinchcomb, A. L., et al. Characterization of the Permeability Barrier of Human Skin *In Vivo*. *Proc. Natl. Acad. Sci. U.S.A.* **1997,** *94*, 1562-1567.

b Romonchuk, W.J.; Bunge, A.L. Mechanism of enhanced dermal permeation of 4-cyanophenol and methyl paraben from saturated aqueous solutions containing both solutes. *Skin Pharmacol. Physiol.* **2010**, *23*, 152–163.

c Cleek, R.L.; Bunge, A.L. A new method for estimating dermal absorption from chemical exposure. 1. General approach. *Pharm. Res.* **1993**, *10*, 497-506.
